# Pathophysiological Mechanisms of Antipsychotic-Induced Parkinsonism

**DOI:** 10.3390/biomedicines10082010

**Published:** 2022-08-18

**Authors:** Elena E. Vaiman, Natalia A. Shnayder, Aiperi K. Khasanova, Anna I. Strelnik, Arseny J. Gayduk, Mustafa Al-Zamil, Margarita R. Sapronova, Natalia G. Zhukova, Daria A. Smirnova, Regina F. Nasyrova

**Affiliations:** 1 V. M. Bekhterev National Medical Research Center for Psychiatry and Neurology, Institute of Personalized Psychiatry and Neurology, 192019 St. Petersburg, Russia; 2 Shared Core Facilities Molecular and Cell Technologies, V.F. Voyno-Yasenetsky Krasnoyarsk State Medical University, 660022 Krasnoyarsk, Russia; 3 International Centre for Education and Research in Neuropsychiatry, Samara State Medical University, 443079 Samara, Russia; 4 Department of Physiotherapy, Faculty of Continuing Medical Education, Peoples’ Friendship University of Russia, 117198 Moscow, Russia; 5 Department of Medical Genetics and Clinical Neurophysiology, Institute of Postgraduate Education, V.F. Voyno-Yasenetsky Krasnoyarsk State Medical University, 660022 Krasnoyarsk, Russia; 6 Department of Neurology and Neurosurgery, Siberian State Medical University, 634050 Tomsk, Russia

**Keywords:** antipsychotic-induced parkinsonism, drug-induced parkinsonism, antipsychotics, theories of pathogenesis, pathophysiology, pathogenesis

## Abstract

Among neurological adverse reactions in patients with schizophrenia treated with antipsychotics (APs), drug-induced parkinsonism (DIP) is the most common motility disorder caused by drugs affecting dopamine receptors. One of the causes of DIP is the disruption of neurotransmitter interactions that regulate the signaling pathways of the dopaminergic, cholinergic, GABAergic, adenosinergic, endocannabinoid, and other neurotransmitter systems. Presently, the development mechanisms remain poorly understood despite the presence of the considered theories of DIP pathogenesis.

## 1. Introduction

Drug-induced parkinsonism (DIP), tardive dyskinesia (TD), tardive dystonia, akathisia, myoclonus, and tremor are drug-induced motility disorders. Among these adverse drug reactions (ADR), DIP is the most widespread movement disorder caused by drugs that antagonise dopamine receptors, after Parkinson’s disease (PD), which has similar clinical manifestations [1,2]. That is why patients with DIP are often misdiagnosed as having PD [1,3]. These patients often took antiparkinsonian drugs for a long period of time, despite the fact that recovery is possible simply by stopping the offending medicinal drugs. Antipsychotics (APs) are the most common cause of DIP. Antipsychotic-induced parkinsonism (AIP) usually manifests itself within a few days or weeks after the start of AP, but in rare cases, the delay in onset can be several months or more [4]. Moreover, the complexity of differential diagnosis arises between DIP and negative symptoms of schizophrenia or withdrawal syndrome, as well as with depression [5].

AIP debuts in patients older than 40 years, women are two times more committed than men [6]. Despite the development of APs of new generations, the problem of AIP has not been solved to date. As shown in Table 1, all APs have a potential risk of AIP ranging from low (+) to high (+++).

**Figure 1:**
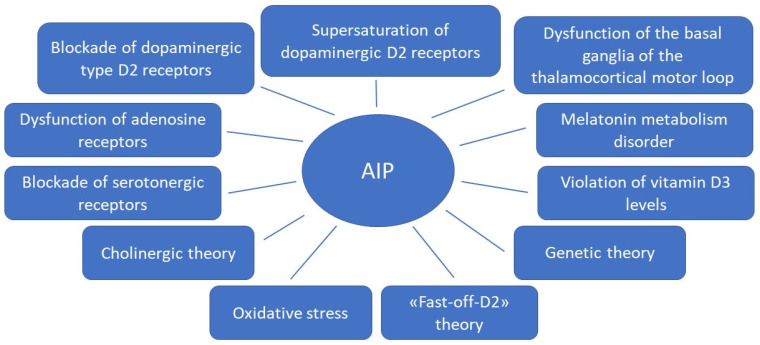
Theories for the development of antipsychotic-induced parkinsonism.

Due to the fact that APs of the first and new generations affect different mechanisms of action in the treatment of schizophrenia spectrum disorders, the risk of developing AIP in some APs of new generations remains high, almost similar to APs of the first generation. This may be due to the fact that the pathophysiological mechanisms of AIP development are more complex than previously thought. In addition to the effect of AP on dopaminergic neurons, other possible mechanisms are being considered (Figure 1). Knowledge of these mechanisms can help in the development of new personalized strategies for the prediction, prevention and early correction of AIP in patients with schizophrenia spectrum, which will improve the therapeutic response and quality of life of the patient. This was the reason for the preparation of this thematic review, the purpose of which is to analyze basic and clinical studies studying on the pathophysiological mechanisms of AIP.

One of the causes of AIP is a disruption of neurotransmitter interactions that regulate the signaling pathways of the dopaminergic, cholinergic, GABA-ergic, adenosinergic, endocannabinoid and other neurotransmitter systems. The monoamine (dopamine, serotonin, and norepinephrine) systems of the brain play an important role in normal behavior, and disturbances in these circuits are thought to be involved in the development of a number of neurological and psychiatric disorders. The dopamine system is involved in the implementation of such brain functions as locomotion, affect and cognition. It is also known that this system is the last monoamine system that is formed in the brain during ontogeny [7], which suggests that it can have an important stabilizing and integrative effect on brain circuits, and that its disruption can cause their dysfunction [8]. Dopamine neurons in the substantia nigra in its dorsal part are associated with the limbic and cortical or associative systems of the brain, while those in the ventral part are associated with motility [9].

### 1.1. Dopamine D2 Type Receptors Blockade

Dopamine receptors in the brain are represented by two families: the D1 (D1 family receptors and D5 receptors), and the D2 (D2, D3 and D4 receptors). All currently available APs are able to antagonize dopamine D2 receptors, and the APs’ therapeutic effects in psychosis are related to their action on the limbic system reducing dopamine transmission. Due to antagonism of dopamine D2 receptors in the striatum, neurons of gamma aminobutyric acid (GABA), encephalin and the subthalamic nucleus are disinhibited at the beginning of the indirect pathway without changing the direct pathway. Due to this, there is an increase in GABA inhibition in the thalamocortical projection by facilitating inhibition in the globus pallidus/reticular substantia nigra. This pathway is similar to the model of basal ganglia motor loop impairment in PD [4]. 

It is assumed that the mechanism of action of APs is associated with the level of occupancy of the dopamine D2 receptors. This is confirmed by several reports that therapeutic doses of typical APs block D2 receptors in 70–89% of cases in young adults, while atypical APs block in 38–63% of cases [10].

### 1.2. Supersaturation (“Occupancies”) of Striatal Dopamine D2/3 Receptors

The exact mechanism of AIP development is still unknown; nevertheless, the main theory is the dopamine receptors blockade. In animal models, about 70% of the occupancy of dopamine D2/3 receptors was recorded during AP therapy, which leads to the development of AIP [11]. The threshold levels of occupancy of dopamine D2/3 receptors in the striatum associated with the development of AIP in young adults, which is about 80%, have been demonstrated in studies using neuroimaging technologies (using positron emission tomography (PET) D2/3 receptor imaging) [12,13]. Dissimilarities in the severity of AIP development are associated with the occupancy density of dopamine D2/3 receptors, AP concentration, the rate of dissociation from the D2 receptor, the selectivity of dopamine receptors in the limbic system and striatum, and the activity of other receptors (for example, serotonergic, muscarinic) [14]. Therefore, typical APs are more associated with an increased risk of developing AIP than atypical APs [14,15,16]. However, such a high occupancy of dopamine receptors should not be considered unequivocally, because the occupancy of receptors is not equal to antagonism. For example, aripiprazole, which, in addition to dopamine receptors, interacts with serotonin (5-HT) types 1A and 2A receptors and rarely causes AIP, even with a dopamine receptor occupancy rate > 95% due to a weak antagonistic effect on dopamine receptors [17,18].

### 1.3. Influence of the Basal Ganglia of the Thalamocortical Motor Loop

**Figure 2:**
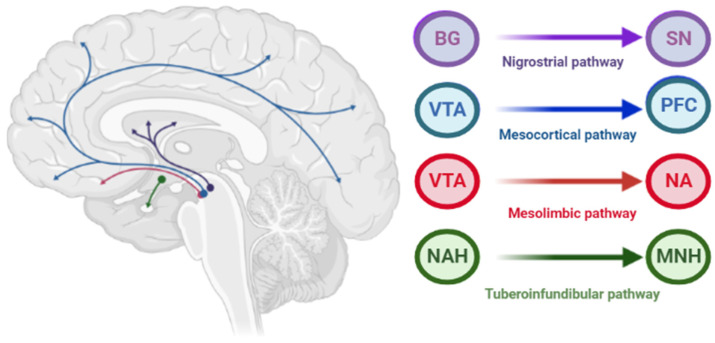
Pathways of dopaminergic neurotransmission. Note: VTA—ventral tegmental area; PFC—prefrontal cortex; NA—nucleus accumbens: SN—substance nigra; BG—basal ganglia (striatum); NAH—nucleus arcuatus (hypothalamus); MNH—middle nucleus (hypothalamus).

The pathophysiology of AIP is associated with drug-induced changes in the motor chain of the basal ganglia secondary to blockade of dopaminergic receptors [4]. The central dopaminergic system is presented of the four pathways: mesolimbic, mesocortical, tuberoinfundibular, and nigrostriatal (Figure 2).

**Figure 3:**
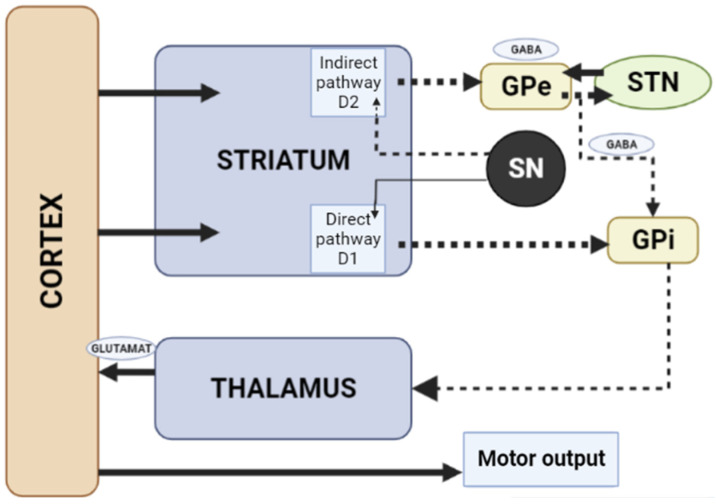
Schematic diagram of the excitatory and inhibitory ganglia involved in the development of AIP (Adapted from [25], Copyright year 2020, BMJ Neurol. Open). Note: Substantia nigra dopaminergic projections exert an exciting effect on stria-pallidal fibers of the direct pathway through dopamine D1 receptors, which leads to disinhibition of the thalamic nuclei and increased thalamocortical excitation, facilitating movements initiated by the cortex. The obstruction of voluntary movement occurs due to thalamic inhibition, due to the inhibition of stria-pallidal fibers in an indirect pathway through dopamine D2 receptors. The direct pathway is due to the activation of glutamate neurons in the sensorimotor cortex, and the indirect pathway is due to the activation of GABA-ergic neurons. The dotted line shows the inhibitory action due to the action of GABA. The straight line shows the excitatory effect due to the action of glutamate.

When dopamine D2 receptors blockade in the striatum, striatal neurons containing GABA and encephalin are disinhibited, affecting the indirect pathway and ultimately leading to a relative decrease in the activity of thalamocortical circuits [4,19]. This effect may be mitigated by APs anticholinergic activity [20,21], as evidenced by the observation that clozapine has a low risk of developing AIP and also has a high relative affinity for muscarinic cholinergic receptors [20]. The reason for the decrease in sufficient concentrations of dopamine in the striatum may also be a decrease in the release of dopamine into the synaptic cleft [21]. Drugs that do not directly affect dopamine levels (valproic acid, calcium channel blockers) can induce DIP through other mechanisms, including modulation of GABA activity or mitochondrial dysfunction (Figure 3) [4,21,22,23,24].

### 1.4. Fast-off-D Theory

In studies devoted to brain occupancy, radioactive clozapine has been proven to show rapid and transient occupancy of the dopamine D2 type receptors, dissociating in less than 60 s after administration, while radioactive haloperidol and chlorpromazine show long-term occupancy with slow dissociation in less than 30 min. Therefore, atypical APs are clinically more effective, having temporary occupancy of D2 type dopamine receptors and rapid dissociation to normal dopamine neurotransmission (Table 2) [26,27].

### 1.5. Role of Adenosine Receptors

Purine and adenosine interact with major neurotransmitter systems (glutamatergic cholinergic, GABA-ergic and dopaminergic) to modulate neuronal function in the central and peripheral nervous systems [28].

Transmission of adenosine occurs through purinergic receptors coupled to the G-protein class P1, which is subdivided into four receptor subtypes: A1, A2A, A2B, and A3 [29]. A2A adenosine receptors are highly expressed by GABAergic neurons in the striatum, globus pallidus, and olfactory bulb [30], and are co-localized with dopamine D2 type receptors in the basal ganglia on enkephalin-expressing output neurons of the indirect pathway leading to the globus pallidus and substantia nigra [31,32], which are in dopaminergic nigrostriatal and mesolimbic neuronal pathways [33]. The A2A and D2 receptors are antagonists and regulate GABA neurons [34,35].

**Figure 4:**
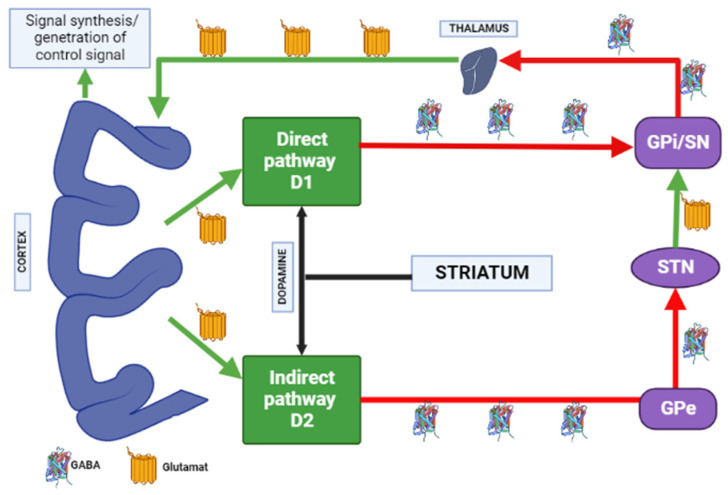
Distribution of A1 and A2A adenosine receptors in the human brain. Note: GPe—globus pallidus external; GPi—globus pallidus internal; STN—subthalamic nucleus; SN—substantia nigra; GABA—gamma aminobutyric acid.

Most evidence suggests that due to intramembrane interaction, activation of the adenosine A2A receptor indirectly blocks the activation of dopamine D2 receptors, and stimulation of D2 receptors blocks the activation of adenylate cyclase caused by the A2A receptor [34]. Upon stimulation of A2A receptors, GABA is released, while upon stimulation of D2 receptors, it is suppressed in the globus pallidus [35,36]. Here is evidence that these receptors, on the contrary, can act as synergists, under certain circumstances (presence of certain isoforms of adenylate cyclase, interruption of previous long-term exposure to D2 receptor agonists), activation of the D2 receptors enhances the effects of A2A receptors [34]. A study by Parsons et al. [37] in rats showed that chronic administration of haloperidol activates striatal A2A receptors. The effect of haloperidol was selective for A2A receptors over other adenosine receptor subtypes. Notably, atypical APs did not affect A2A receptors’ density in this study [37]. A2A adenosine receptor antagonists suppress motor disorders such as catalepsy and locomotion induced by dopamine antagonists [38,39]. A2A receptor antagonists are effective in relieving muscle rigidity and tremor in AIP (Figure 4) [15,40,41].

### 1.6. Blockade of the Serotonergic System

The serotonergic system plays a crucial role in various physiological actions regulation, including psychoemotional, cognitive, sensorimotor, and autonomic functions [42,43,44]. Serotonergic neurotransmission is presented by several 5-HT receptors, which are classified into 7 families (5-HT1 to 5-HT7) and 14 subtypes (5-HT1A, 1B, 1D, 1E, 1F, 5-HT2A, 2B, 2C, 5-HT4, 5-HT3, 5-HT5A, 5B, 5-HT6 and 5-HT7) [45,46]. The 5-HT1E, 5-HT1F, and 5-HT5 receptors are associated with the Gi/o protein and inhibit adenylate cyclase activity, cyclic adenosine monophosphate (cAMP) formation, and protein kinase A (PKA) activity. The 5-HT2A, 5-HT2B, and 5-HT2C receptors are coupled to the Gq protein and increase phosphatidylinositol (PI) turnover by activating phospholipase C, thereby stimulating the protein kinase C^−^ and Ca^2+^/calmodulin cascade. The 5-HT4, 5-HT6, and 5-HT7 receptors are Gs-coupled and activate adenylate cyclase and PKA [44,45,46].

**Figure 5:**
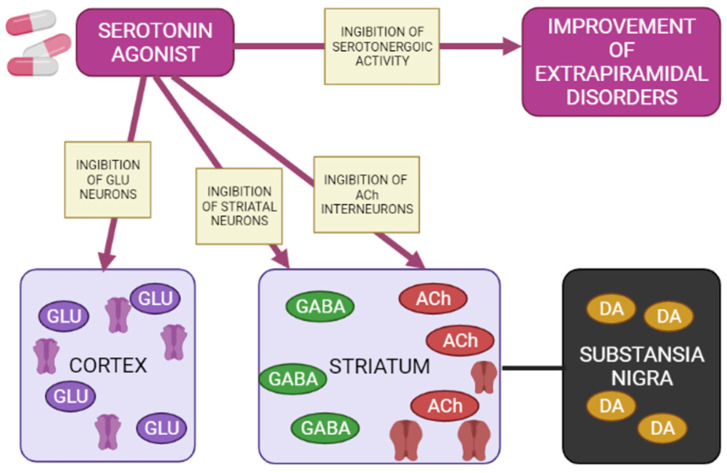
Mechanisms of action of a 5-HT1A agonist in modulating extrapyramidal motility disorders (Adapted from [65], Copyright year 2012, Adv. Biol. Psychiatry). Note: GLU—glutamate; GABA—gamma aminobutyric acid; Ach—acetylcholine; DA—dopamine.

5-HT1A receptors are localized in the raphe nucleus, hippocampus, amygdala, and lateral septum. Expression of 5-HT1A receptors occurs in the cerebral cortex, basal ganglia (striatum), and diencephalon (thalamus and hypothalamus) in low to moderate density [42,47,48]. Inhibition of adenylate cyclase activity by 5-HT1A receptors leads to inhibition of the cAMP-PKA cascade. In addition, 5-HT1A receptors activate G-protein-gated inwardly rectifying potassium channels (GIRK), which hyperpolarize target neurons and suppress their activity [41,43,44,45]. Several studies on animal models have shown that 5-HT1A receptors are activated with the introduction of AP for dehydration, has a protective effect on the development of extrapyramidal syndrome (EPS) [49,50,51,52,53,54,55]. The 5-HT2A and 5-HT2C receptors are highly expressed in the cerebral cortex, olfactory tubercle and limbic system (nucleus accumbens, hippocampus), basal ganglia (striatum and substantia nigra). 5-HT2A/2C antagonists attenuate AP-induced EPS in PD patients [56,57,58] by increasing the level of released acetylcholine, dopamine metabolism, and Fos protein expression in the striatum [57]. 5-HT3 receptors constitute a heteropentamer consisting of subunits from 5-HT3A to 5-HT3E and function as a cation (Na^+^, K^+^, and Ca^2+^) permeable channels [45,59]. Therefore, when 5-HT3 receptors are activated, postsynaptic membranes are depolarized, thereby exciting target neurons. Serotonin 5-HT3 receptors are located in the nerve endings of various neurons and induce the release of neurotransmitters (acetylcholine, glutamate, GABA and dopamine). Clinical studies have also shown that 5-HT3 receptor antagonists significantly reduce the frequency and severity of AP-induced EPS (e.g., AIP) in patients with chronic schizophrenia [60,61]. However, a recent study showed that serotonin 5-HT3 receptors do not affect the activity of cholinergic interneurons in the striatum [62]. Thus, the functional mechanisms of 5-HT3 receptors in EPS are still unclear. The expression of serotonin 5-HT6 receptors mainly occurs in the brain, in particular, in the basal ganglia (striatum and nucleus accumbens), limbic system (olfactory tubercles and hippocampus), and cerebral cortex [45]. According to the results of clinical studies, 5-HT6 receptor antagonists also have a protective property against the development of EPS [63,64]. The decrease in the incidence and severity of EPS in the presence of 5-HT6 antagonists was further confirmed by an electrophysiological study [62], reflecting a decrease in the activation of striatal acetylcholine neurons, thereby reducing the likelihood of developing EPS (Figure 5) [65].

Agonists of postsynaptic and presynaptic serotonin 5-HT1A receptors lead to a de-crease in the manifestations of extrapyramidal movement disorders. This is explained by hyperpolarization GABA medial spine neurons or indirectly by inhibition acetylcholinergic and glutamatergic interneurons in the striatum. In addition, when presynaptic 5-HT1A autoreceptors are stimulated, the serotonergic activity of neurons in the nuclei of the sutures is inhibited, reducing the functions of 5-HT2A/2C, 5-HT3 and 5-HT6 receptors, as a result of which the symptoms of extrapyramidal movement disorders improve. 

### 1.7. Cholinergic Theory

The desired blockade of the mesolimbic pathways leads to simultaneous inhibition of dopamine D2 type receptors in the substantia nigra, resulting in AIP. Cholinergic interneurons in the basal ganglia balance dopaminergic activity. Accordingly, in PD, when the balance is disturbed due to the degeneration of dopaminergic neurons, there is a relative predominance of cholinergic activity. Thus, the pathogenesis in PD can be considered as both a lack of dopaminergic activity and a relative increase in cholinergic activity.

Based on this theory, cholinergic drugs can also be prescribed to patients in order to restore the balance of the dopaminergic and cholinergic systems [66].

### 1.8. Melatonin Theory

Melatonin interacts with 5-HT2 receptors and can regulate the activity of dopamine. In case of higher concentrations, melatonin acts as a 5-HT2 receptor antagonist and leads to an increase in dopaminergic activity, while at physiological concentrations, melatonin reduces dopamine, acting as an agonist. These modulating effects of melatonin on dopamine activity may explain the circadian changes in dopamine activity [67,68] and the circadian changes in the clinical course in patients with schizophrenia treated with APs; as a result, a reduction in the symptom severity can be expected [69]. Animal studies indicate that APs interfere with melatonin secretion. In rats, APs sharply increase melatonin levels in the pineal gland and blood plasma [70]. Similarly, in humans, plasma melatonin levels increase during AP therapy [71]. As a result of antagonism of the 5-HT2 receptor by high concentrations of melatonin, dopamine activity will increase, thereby counteracting the blockade of dopamine receptors caused by APs. Thus, the interaction between the decrease in dopamine activity during the use of APs, and the protective effect of therapeutic concentrations of melatonin on dopamine function in the form of its increase, may partially explain the delay in AP effects, the delayed appearance of AIP, and possibly the regression of AIP. However, because chronic use of APs (for more than 6 months) can eventually damage the cells of the pineal gland [72], it can be expected that melatonin secretion will decrease, which will contribute to ADRs in the background of AP. In confirmation of this theory, clinical examples are presented, where patients with AIP risk factors associated with reduced melatonin secretion (older age, postmenopausal women, depression [73]) had a history of AIP more often [74,75,76,77].

### 1.9. Theory of Oxidative Stress

The theory of oxidative stress is actively studied in the pathogenesis of extrapyramidal movement disorders. Despite the fact that damage to cellular structures is observed during oxidative stress, its components act as second messengers in the formation of regulatory functions. One of the free radicals of oxidative stress that regulates various cellular signaling pathways is nitric oxide (NO) [78]. When NO synthase is inhibited, enzyme activity is reduced by 80%, leading to the formation of primary and secondary products of lipid peroxidation (conjugated dienes (CD) and trienes (CT), malondialdehyde (MDA) and Schiff bases (SB)). According to the study by Dolgo-Saburov et al. [79], a significant increase in the concentration of CD and CT is observed after blockade of the dopamine D2 type receptors while taking APs in the studied structures of the central nervous system. The accumulation of MDA and SB also indicates the development of oxidative stress and its maintenance after a decrease in NO synthase activity [79]. Haloperidol-induced AIP is associated with elevated levels of the haloperidol metabolite, pyridinium, which is toxic to dopamine neurons [80,81]. This toxic metabolite can be produced locally in the brain [82]. During haloperidol therapy, oxidative metabolism in the brain increases [83], protein kinase B (Akt) phosphorylation decreases, which leads to caspase-3 activation [84]. Haloperidol alters the expression of the transcription nuclear factor kappa B (NF-κB) in the substantia nigra, which directly affects oxidative stress [85] and plays a role in the activation of several genes involved in the immune and inflammatory systems [86].

### 1.10. Role of Vitamin D3

The vitamin D3 receptor (VDR) is a widespread steroid receptor, mainly localized in the pigmented nigrostriatal tract and motor cortex [87,88], providing melanin synthesis, cytoskeletal stability, and calcium in the motor area [89]. Indirectly, the control of calcium during the inhibition of NMDA receptors (NMDAR) in the elimination of excitotoxicity in vitro and in vivo has been described [90,91]. In addition, NMDAR enhancement is involved in glutamate excitotoxicity, degeneration in the nigrostriatal tract and motor cortex by increasing inward calcium flow, thereby increasing the level of calcium in the brain [92]. Activation of VDR by its agonists and inhibition of NMDAR in vitro reduces calcium toxicity and stabilizes microtubules, with a protective effect against excitotoxicity and synaptic denervation [93,94,95]. Reducing the reversibility of cortical degeneration (parkinsonism) with calcium supplementation mediated by VDR and NMDAR is still relatively unexplored [96]. Potentiation of VDR and inhibition of NMDAR reduce the level of toxicity in the cerebral cortex by improving neuronal metabolism, the amount of glia and the cytoskeleton to support synaptic function, thus increasing neural activity and motor-cognitive functions. Against the background of taking AP, synaptic denervation occurs due to a calcium-dependent depolymerization of microtubules in motor neurons [97]. It also increases the activity of glia and cells, leading to inflammation [98,99]. Motor ADR in the presence of APs may be the result of antagonism of D2 type dopamine receptors, resulting in the induction of oxidative stress and increased production of free radicals (ROS) [100] due to which the concentration of calcium ions increases both intracellularly and extracellularly [101,102]. This accumulation of calcium leads to microtubule collapse and excessive phosphorylation of the microtubule-associated protein tau, which is involved in the stabilization of the structure of axons and dendrites [103,104]. Recent studies have established an interaction between elevated calcium levels, autophagy, and synaptic denervation in the pathogenesis of AIP [90,91,105]. Elevated calcium and glutamate concentrations, combined with low serum vitamin D3 levels, are associated with the risk of developing parkinsonism and other neurodegenerative diseases [106,107,108].

### 1.11. Genetic Theory

The role of genetic risk factors in the mechanisms of development of AIP [109] has been known for more than 40 years. This was also confirmed by later studies [110,111,112]. Individual differences in susceptibility to haloperidol-induced parkinsonism were explained by individual pharmacokinetic variability associated with the influence of carriage of single nucleotide variants (SNVs) of the *CYP3A4* and *CYP3A5* genes, which affects the metabolism of haloperidol to its pyridinium ion. While taking haloperidol, pyridinium ion-related toxicity may be observed, which may be exacerbated in patients on polytherapy with antidepressants, as they interact with this system [6,86,113,114,115,116].

**Figure 6:**
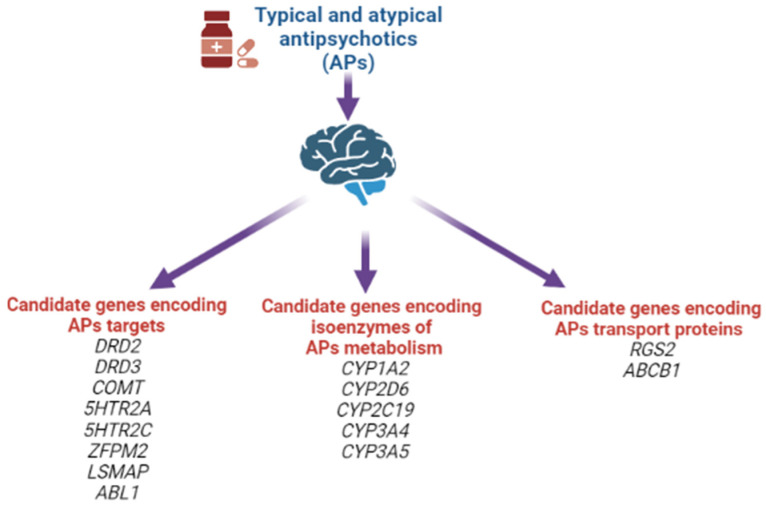
Candidate genes for the risk of developing antipsychotic-induced parkinsonism.

Moreover, at the moment, other associations of candidate genes for the risk of developing AIP have already been studied, shown in Figure 6.

In our systematic review, the following positive associations for the risk of developing AIP were noted: rs1799732 (NG_008841.1:g.4750dup), rs1800497 (NG_012976.1:g.17316G>A), rs6275 (NG_008841.1:g.67525T>C) of the *DRD2* gene; rs167771 (NG_008842.2:g.46980C>T, NG_008842.2:g.46980C>G, NG_008842.2:g.46980C>A) of the *DRD3* gene; rs4680 (NG_011526.1:g.27009G>A) of the *COMT* gene; rs6311 (NG_013011.1:g.4692G>T, NG_013011.1:g.4692G>A) of the *5HTR2A* gene; rs6318 (NG_012082.2:g.152242G>C, NG_012082.2:g.152242G>T) and rs3813929 (NG_012082.2:g.4963C>G, NG_012082.2:g.4963C>T) of the *HTR2C* gene; rs2179652 (NG_022822.1:g.225C>T), rs2746073 (NG_012800.1:g.6059T>A), rs4606 (NG_012800.1:g.8004C>G, NG_012800.1:g.8004C>T), rs1152746 (NC_000001.11:g.192827775C>G, NC_000001.11:g.192827775C>T), rs1819741 (NC_000001.11:g.192815708T>A, NC_000001.11:g.192815708T>A) and rs1933695 (NC_000001.11:g.192795690G>A) of the *RGS2* gene; rs4795390 (NG_030330.1:g.3434G>A, NG_030330.1:g.3434G>C, NG_030330.1:g.3434G>T) of the *PPP1R1B* gene; rs6265 (NG_011794.1:g.68690G>A) of the *BDNF* gene; rs12678719 (NG_011723.2:g.189908C>G) of the *ZFPM2* gene; rs938112 (NC_000003.12:g.117129003C>A, NC_000003.12:g.117129003C>T) of the *LSMAP* gene; rs2987902 (NG_012034.1:g.125979A>T) of the *ABL1* gene. However, at present, it should be recognized that there is no definitive or single decision on the leading role of any particular SNVs/polymorphisms in AIP development [6,114,116,117].

## 2. Discussion

**Figure 7:**
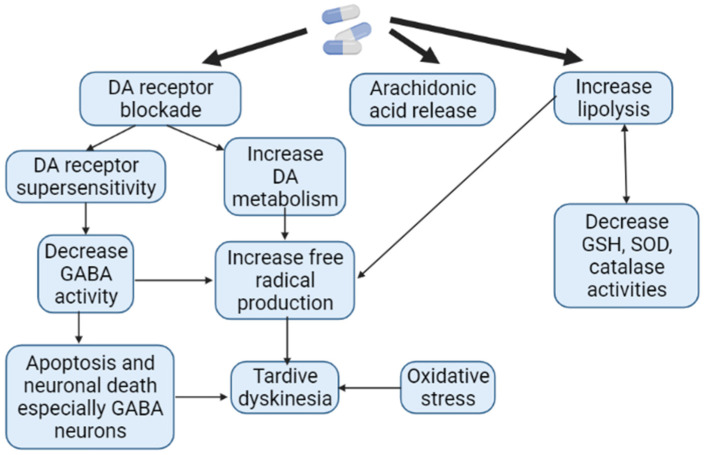
Pathogenesis of antipsychotic-induced tardive dyskinesia (Adapted from [119], Copyright year 2016, J. Exp. Pharmacol): Note: DA—dopamine; GABA—gamma aminobutyric acid; GSH—glutathione; SOD—superoxide dismutase.

In terms of motor function disorders, AIP and AP-induced TD are the most common neurological ADRs associated with APs use. However, to the present date, it remains unclear which patient and against the background of taking which specific AP one or another ADRs will develop. The pathophysiological mechanisms of AIP and AP-induced TD are different. While there is a decrease in the function of the extrapyramidal system in the form of the development of bradykinesia, muscle rigidity in AIP, in AP-induced TD, on the contrary, there is an increase in function in the form of violent movements [118]. It has been shown that long-term use of AP is associated with the formation of ROS as a result of increased metabolism of catecholamines (Figure 7). The reduction in such oxidative damage to AP-sensitive dopaminergic neurons has become the basis of antioxidant treatment for AP-induced TD. However, the effectiveness of AIP antioxidant therapy is low. While AIP results from the interaction of APs with substantia nigra neurons, dyskinetic symptoms may also be more closely related to AP-induced problems in the striatum, in particular the caudate nucleus [23,66].

It should be assumed that the development of AIP in patients with schizophrenia is influenced by such risk factors as taking APs of the first generation (in 50% of patients AIP eventually develops while taking APs); elderly age; genetic predisposition; a history of human immunodeficiency virus infection. On the other hand, when predicting the development of AIP, one should take into account not only the risk factors, but also protective factors of AIP: taking anticholinergic drugs (due to increased cholinergic activity, which leads to stimulation of the GABA-ergic inhibitory pathway in the basal ganglia); smoking (nicotine can act as an inhibitor of monoamine oxidase B, increasing the availability of dopamine) [28]. In recent decades, genetic predictors of AIP and AP-induced TD have been actively studied as a risk for the development of these neurological ADRs [120,121,122].

The theories of AIP development discussed in this review are very important. First of all, knowledge about the AIP pathogenesis can help with better understanding of the disease nature. Moreover, thanks to the understanding of the pathogenetic mechanisms of AIP, new opportunities for the treatment of this ADR are opening up. At the same time, knowledge about the pathogenesis of AIP can help to develop personalized strategies for the correction of patients suffering from AIP or at risk of developing AIP. Despite the large number of theories presented, the pathogenetic mechanism of this neurological ADR remains unclear.

Thus, it is impossible to single out one of the theories as the dominant mechanism for the development of AIP. Undoubtedly, the pathophysiology of AIP should be considered as an interconnected complex of neurotransmitter systems (dopamine, serotonin, adenosine, acetylcholine, and others) that affect each other and depend on nonmodifiable risk factors—gender, age, genetic predisposition—as well as modifiable ones—the type of AP, its dose, concomitant diseases, etc. At the same time, knowledge of this raises questions about the personalized risks of developing AIP in a particular patient because the pathophysiological mechanisms of developing AIP may differ in different patients. Thus, this poses new challenges and requires additional research to better understand the molecular pathophysiology of AIP.

The limitations for this review were the difficulties in finding full-text publications and the presence of abstracts that did not reveal the full information of the theories of pathogenesis. Moreover, in many similar works, information is duplicated in a compressed version. For a complete understanding of the pathogenetic mechanisms, it would be interesting to consider the differences, taking into account the gender and age of patients with AIP. We did not find any articles with reliable data assessing differences by sex and age in this review, which are the predictors of the risk of developing AIP. Thus, a review of these theories is relevant, but we have not found such works.

In this review, we have described the pathogenetic mechanisms of AIP development found in the literature and presented the theories in as much detail as possible with the help of figures and tables for better understanding.

## 3. Conclusions

Our review of studies of the mechanisms of AIP development in an animal model and patients with schizophrenia allows us to identify the following theories: impaired function of D2 type dopamine receptors (blockade of D2 type dopamine receptors, supersaturation of D2 type dopamine receptors, “Fast-off-D2” theory); dysfunction of the basal ganglia of the thalamocortical motor loop; dysfunction of adenosine receptors; blockade of serotonergic receptors; cholinergic theory; oxidative stress; violation of melatonin metabolism; violation of the level of vitamin D3; genetic theory. The search for the leading pathophysiological mechanisms of AIP is important for the development of a personalized strategy, prevention, therapy, considered neurological ADRs of AP-therapy in real clinical practice.

## Figures and Tables

**Table 1:** The risk of developing parkinsonism induced by taking antipsychotics of the first and new generations.

Antipsychotics of First Generation	Risk of Developing AIP	Antipsychotics of New Generations	Risk of Developing AIP
Haloperidol	+++	Lurasidone	++
Pimozide	+++	Olanzapine	++
Thiothixene	+++	Paliperidone	++
Fluphenazine	+++	Risperidone	++
Loxapin	++	Azenapine	+
Molindon	++	Aripiprazole	+
Perphenazine	++	Brexpiprazole	+
Trifluoperazine	++	Ziprasidone	+
Chlorpromazine	++	Iloperidone	+
Thioridazine	+	Karipazin	+
		Quetiapine	+
		Clozapine	+
		Lumateperone	+
		Pimavenzirin	+

Note: + low risk; ++ moderate risk; +++ high risk.

**Table 2:** Dopamine receptors occupancy while taking antipsychotics (Adapted from [27], Copyright year 2013 Neurosci. J.).

Antipsychotics	Occupancy Percentage (%)		
	D2 Receptors	D3 Receptors	D4 Receptors
Clozapine	38–63	62	49–73
Chlorpromazine	78	62	17
Haloperidol	85	52	57
Olanzapine	43–89	10–55	27–80
Risperidone	63–89	25–61	22–55
Quetiapine	51	24	88

## Data Availability

Not applicable.

## References

[1] Miller L.G., Jankovic J. (1990). Neurologic approach to drug-induced movement disorders: A study of 125 patients. South. Med. J..

[2] Sethi K.D. (2001). Movement disorders induced by dopamine blocking agents. Semin. Neurol..

[3] Esper C.D., Factor S.A. (2008). Failure of recognition of drug-induced parkinsonism in the elderly. Mov. Disord..

[4] Shin H.W., Chung S.J. (2012). Drug-induced parkinsonism. J. Clin. Neurol..

[5] Caroff S.N., Hurford I., Lybrand J., Campbell E.C. (2011). Movement disorders induced by antipsychotic drugs: Implications of the CATIE schizophrenia trial. Neurol. Clin..

[6] Shnayder N.A., Vaiman E.E., Neznanov N.G., Nasyrova R.F. (2022). Pharmacogenetics of Antipsychotic-Induced Extrapyramidal Disorders.

[7] Lauder J.M., Bloom F.E. (1974). Ontogeny of monoamine neurons in the locus coeruleus, Raphe nuclei and substantia nigra of the rat. I. Cell differentiation. J. Comp. Neurol..

[8] Grace A.A. (2016). Dysregulation of the dopamine system in the pathophysiology of schizophrenia and depression. Nat. Rev. Neurosci..

[9] Lynd-Balta E., Haber S.N. (1994). The organization of midbrain projections to the striatum in the primate: Sensorimotor-related striatum versus ventral striatum. Neuroscience..

[10] Nyberg S., Dencker S.J., Malm U., Dahl M.-L., Svetnson J.-O., Halldin C., Naskashima Y., Farde L. (1998). D(2)- and 5-Ht(2) receptor occupancy in high-dose neuroleptictreated patients. Int. J. Neuropsychopharmacol..

[11] Crocker A.D., Hemsley K.M. (2001). An animal model of extrapyramidal side effects induced by antipsychotic drugs: Relationship with D2 dopamine receptor occupancy. Prog. Neuro-Psychopharmacol. Biol. Psychiatry.

[12] Farde L., Nordstrom A.L., Wiesel F.A., Pauli S., Halldin C., Sedvall G. (1992). Positron emission tomographic analysis of central D1 and D2 dopamine receptor occupancy in patients treated with classical neuroleptics and clozapine. Relation to extrapyramidal side effects. Arch. Gen. Psychiatry.

[13] Scharrer J., Tatsch K., Schwarz J., Oertel W.H., Konjarczyk M., Albus M. (1994). D2-dopamine receptor occupancy differs between patients with and without extrapyramidal side effects. Acta Psychiatr. Scand..

[14] Haddad P.M., Dursun S.M. (2008). Neurological complications of psychiatric drugs: Clinical features and management. Hum. Psychopharmacol..

[15] Margolese H.C., Chouinard G., Kolivakis T.T., Beauclair L., Miller R. (2005). Tardive dyskinesia in the era of typical and atypical antipsychotics. Part 1: Pathophysiology and mechanisms of induction. Can. J. Psychiatry..

[16] Sebastiao A.M., Ribeiro J.A. (2009). Adenosine receptors and the central nervous system. Handb. Exp. Pharmacol..

[17] Grunder G., Carlsson A., Wong D.F. (2003). Mechanism of new antipsychotic medications: Occupancy is not just antagonism. Arch. Gen. Psychiatry.

[18] Sharma A., Sorrell J.H. (2006). Aripiprazole-induced parkinsonism. Int. Clin. Psychopharmacol..

[19] Gunne L.M., Andrén P.E. (1993). An animal model for coexisting tardive dyskinesia and tardive parkinsonism: A glutamate hypothesis for tardive dyskinesia. Clin. Neuropharmacol..

[20] Snyder S., Greenberg D., Yamamura H.I. (1974). Antischizophrenic drugs and brain cholinergic receptors. Affinity for muscarinic sites predicts extrapyramidal effects. Arch. Gen. Psychiatry..

[21] Susatia F., Fernandez H.H. (2009). Drug-induced parkinsonism. Curr. Treat. Options Neurol..

[22] Ward K.M., Citrome L. (2018). Antipsychotic-Related Movement Disorders: Drug-Induced Parkinsonism vs. Tardive Dyskinesia-Key Differences in Pathophysiology and Clinical Management. Neurol. Ther..

[23] Vaiman E.E., Shnayder N.A., Neznanov N.G., Nasyrova R.F. (2019). Pathophysiological mechanisms underlying antipsychotic-induced tardive dyskinesia. Bull. Sib. Med..

[24] Ossowska K. (2002). Neuronal basis of neuroleptic-induced extrapyramidal side effects. Pol. J. Pharmacol..

[25] Powell A., Gallur L., Koopowitz L., Hayes M.W. (2020). Parkinsonism in the psychiatric setting: An update on clinical differentiation and management. BMJ Neurol. Open.

[26] Kapur S., Seeman P. (2001). Does fast dissociation from the dopamine D2 receptor explain the action of atypical antipsychotics? A new hypothesis. Am. J. Psychiatry.

[27] Bohlega S.A., Al-Foghom N.B. (2013). Drug-induced Parkinson’s disease. A clinical review. Neurosci. J..

[28] Fuxe K., Ferré S., Canals M., Torvinen M., Terasmaa A., Marcellino D., Goldberg S.R., Staines W., Jacobsen K.X., Lluis C. (2005). Adenosine A2A and dopamine D2 heteromeric receptor complexes and their function. J Mol. Neurosci..

[29] Kulisevsky J., Poyurovsky M. (2012). Adenosine A2A-receptor antagonism and pathophysiology of Parkinson’s disease and drug-induced movement disorders. Eur. Neurol..

[30] Jenner P., Mori A., Hauser R., Morelli M., Fredholm B., Chen J. (2009). Adenosine, adenosine A 2A antagonists, and Parkinson’s disease. Parkinsonism Relat. Disord..

[31] Hettinger B.D., Lee A., Linden J., Rosin D.L. (2001). Ultrastructural localization of adenosine A2A receptors suggests multiple celular sites for modulation of GABAergic neurons in rat striatum. J. Comp. Neurol..

[32] Varty G.B., Hodgson R.A., Pond A.J., Grzelak M.E., Parker E.M., Hunter J.C. (2008). The effects of adenosine A2A receptor antagonists on haloperidol-induced movement disorders in primates. Psychopharmacology.

[33] Müller C.E., Ferre S. (2007). Blocking striatal adenosine A 2A receptors: A new strategy for basal ganglia disorders. Recent Pat. CNS Drug Discov..

[34] Dayne M.R., Larson G., Orona R.A., Zahniser N.R. (1996). Opposing actions of adenosine A2a and dopamine D2 receptor activation on GABA release in the basal ganglia: Evidence for an A2a/D2 receptor interaction in globus pallidus. Synapse.

[35] Ferre S., Fredholm B.B., Morelli M., Popoli P. (1997). Adenosine-dopamine receptor- receptor interactions as an integrative mechanism in the basal ganglia. Trends Neurosci..

[36] Parsons B., Togasaki D.M., Kassir S., Przedborski S. (1995). Neuroleptics up-regulate adenosine A 2a receptors in rat striatum: Im- 487 plications for the mechanism and the treatment of tardive dyskinesia. J. Neurochem..

[37] Bishnoi M., Chopra K., Kulkarni S.K. (2006). Involvement of adenosinergic receptor system in an animal model of tardive dyskinesia and associated behavioural, biochemical and neurochemical changes. Eur. J. Pharmcol..

[38] Bishnoi M., Chopra K., Kulkarni S.K. (2007). Theophylline, adenosine receptor antagonist prevents behavioral, biochemical and neurochemical changes associated with an animal model of tardive dyskinesia. Pharmacol. Rep..

[39] Wardas J., Konieczny J., Lorenc-Koci E. (2001). SCH 58261, an A(2A) adenosine receptor antagonist, counteracts parkinsonian-like muscle rigidity in rats. Synapse.

[40] John D.S., Salamone J.D., Betz A.J., Ishiwari K., Felsted J., Madson L., Mirante B., Clark K., Font L., Korbey S. (2008). Tremorolytic effects of adenosine A2A antagonists: Implications for parkinsonism. Front. Biosci..

[41] Roth B.L. (1994). Multiple serotonin receptors: Clinical and experimental aspects. Ann. Clin. Psychiatry..

[42] Baumgarten H.G., Grozdanovic Z. (1995). Psychopharmacology of central serotonergic systems. Pharmacopsychiatry.

[43] Ohno Y. (2011). Therapeutic role of 5-HT1A receptors in the treatment of schizophrenia and Parkinson’s disease. CNS Neurosci. Ther..

[44] Barnes N.M., Sharp T. (1999). A review of central 5-HT receptors and their function. Neuropharmacology.

[45] Ohno Y., Tatara A., Shimizu S., Sasa M., Sumiyoshi T. (2012). Management of Cognitive Impairments in Schizophrenia: The Therapeutic Role of 5-HT Receptors. Schizophrenia Research: Recent Advances.

[46] Ohno Y., Shimizu S., Imaki J., Masui A., Tatara A., Schwartz T.L., Topel M., Menga J.L. (2013). Management of Antipsychotic-Induced Extrapyramidal Motor Disorders: Regulatory Roles of the Serotonergic Nervous System. Antipsychotic Drugs: Pharmacology, Side Effects and Abuse Prevention.

[47] Pucadyil T.J., Kalipatnapu S., Chattopadhyay A. (2005). The serotonin1A receptor: A representative member of the serotonin receptor family. Cell. Mol. Neurobiol..

[48] Neal-Beliveau B.S., Joyce J.N., Lucki I. (1993). Serotonergic involvement in haloperidol-induced catalepsy. J. Pharmacol. Exp. Ther..

[49] Wadenberg M.L., Young K.A., Richter J.T., Hicks P.B. (1999). Effects of local application of 5-hydroxytryptamine into the dorsal or median raphe nuclei on haloperidol-induced catalepsy in the rat. Neuropharmacology.

[50] Mignon L., Wolf W.A. (2002). Postsynaptic 5-HT1A receptors mediate an increase in locomotor activity in the monoamine-depleted rat. Psychopharmacology.

[51] Ohno Y., Shimizu S., Imaki J. (2008). Evaluation of the antibradykinetic actions of 5-HT1A agonists using the mouse pole test. Prog. Neuro-Psychopharmacol. Biol. Psychiatry.

[52] Ohno Y., Shimizu S., Imaki J. (2008). Anticataleptic 8-OH-DPAT preferentially counteracts with haloperidol-induced Fos expression in the dorsolateral striatum and the core region of the nucleus accumbens. Neuropharmacology.

[53] Ohno Y., Shimizu S., Imaki J. (2009). Effects of tandospirone, a 5-HT1A agonistic anxiolytic agent, on haloperidol-induced catalepsy and forebrain Fos expression in mice. J. Pharmacol. Sci..

[54] Shimizu S., Tatara A., Imaki J., Ohno Y. (2010). Role of cortical and striatal 5-HT1A receptors in alleviating antipsychotic-induced extrapyramidal disorders. Prog. Neuro-Psychopharmacol. Biol. Psychiatry.

[55] Meltzer H.Y. (1991). The mechanism of action of novel antipsychotic drugs. Schizophr. Bull..

[56] Ohno Y., Ishida-Tokuda K., Ishibashi T. (1997). Potential role of 5-HT2 and D2 receptor interaction in the atypical antipsychotic action of the novel succimide derivative, perospirone. Pol. J. Pharmacol..

[57] Kapur S., Remington G. (2001). Atypical antipsychotics: New directions and new challenges in the treatment of schizophrenia. Annu. Rev. Med..

[58] Livesey M.R., Cooper M.A., Deeb T.Z. (2008). Structural determinants of Ca^2+^ permeability and conduction in the human 5-hydroxytryptamine type 3A receptor. J. Biol. Chem..

[59] Zhang Z.J., Kang W.H., Li Q. (2006). Beneficial effects of ondansetron as an adjunct to haloperidol for chronic, treatment-resistant schizophrenia: A double-blind, randomize, placebo-controlled study. Schizophr. Res..

[60] Akhondzadeh S., Mohammadi N., Noroozian M., Karamghadiri N., Ghoreishi A., Jamshidi A.-H., Forghani S. (2009). Added ondansetron for stable schizophrenia: A double blind, placebo controlled trial. Schizophr. Res..

[61] Bonsi P., Cuomo D., Ding J., Sciamanna G., Ulrich S., Tscherter A., Bernardi G., Surmeier D.J., Pisani A. (2007). Endogenous serotonin excites striatal cholinergic interneurons via the activation of 5-HT2C, 5-HT6, and 5-HT7 serotonin receptors: Implications for extrapyramidal side effects of serotonin reuptake inhibitors. Neuropsychopharmacology.

[62] Ohno Y., Imaki J., Mae Y. (2011). Serotonergic modulation of extrapyramidal motor disorders in mice and rats: Role of striatal 5-HT3 and 5-HT6 receptors. Neuropharmacology.

[63] Tatara A., Shimizu S., Shin N., Sato M., Sugiuchi T., Imalki J., Ohno Y. (2012). Modulation of antipsychotic-induced extrapyramidal side effects by medications for mood disorders. Prog. Neuro-Psychopharmacol. Biol. Psychiatry.

[64] Ohno Y., Shimizu S., Tokudome K. (2013). Pathophysiological roles of serotonergic system in regulating extrapyramidal motor functions. Biol. Pharm. Bull..

[65] Ebmeier K.P., O’Brien J.T., Taylor J.-P. (2012). Psychiatry of Parkinson’s Disease. Adv. Biol. Psychiatry.

[66] Naber D., Wirz-Justice A., Kafka M.S. (1981). Seasonal variations in the endogenous rhythms of dopamine receptor binding in the rat striatum. Biol. Psychiatry.

[67] Naber D., Wirz-Justice A., Kafka M.S. (1982). Chronic fluphenazine treatment modifies circadian rhythms of neurotransmitter receptor binding in the rat. J. Neural Transm..

[68] Brown W.A., Herz L.R. (1989). Response to neuroleptic drugs as a device for classifying schizophrenia. Schizophr. Bull..

[69] Gaffori O., Geffard M., Van Ree J.M. (1983). des-Tyr1-gamma-endorphin and haloperidol increase pineal gland melatonin levels in rats. Peptides.

[70] Smith J.A., Mee T.J.X., Barnes J.D. (1978). Increased serum melatonin levels in chlorpromazine-treated psychiatric patients. J. Neural Transm..

[71] Horita N., Ishii T., Moroji T. (1978). Effects of long-term administration of chlorpromazine on the pineal gland of rats. Acta Neuropathol..

[72] Ayd F.U. (1961). A survey of drug-induced extrapyramidal reactions. J. Am. Med. Assoc..

[73] Sack R.L., Lewy A.J., Erb D.L., Vollmer W.M., Singer C.M. (1986). Human melatonin production decreases with age. J. Pineal Res..

[74] Miles A., Philbrick D.R.S. (1988). Melatonin and psychiatry. Biol. Psychiatry.

[75] Trentini G.P., De Gaetani C.F., Criscuolo M., Migaldi M., Ferrari G., Trentini G.P., De Gaetani C., Pevet P. (1987). Pineal Calcification in Different Physiopathological Conditions in Humans. Fundamentals and Clinics in Pineal Research.

[76] Sandyk R., Kay S.R., Gillman M.A. (1992). The role of melatonin in the antipsychotic and motor-side effects of neuroleptics: A hypothesis. Int. J. Neurosci..

[77] Zenkov N.K., Lankin V.Z., Men’shikova E.B. (2001). Oxidative Stress: Biochemical and Pathophysiological Aspects.

[78] Dolgo-Saburov V.B., Dagaev S.G., Kubarskaya L.G., Solovjeva N.E. (2012). The role of the nitric oxide generation system in neuro lepticinduced Parkinsonism. Dokl. Biol. Sci..

[79] Iwahashi K., Anemo K., Nakamura K., Fukunishi I., Igarashi K. (2001). Analysis of the metabolism of haloperidol and its neurotoxic pyridinium metabolite in patients with drug-induced parkinsonism. Neuropsychobiology.

[80] Ulrich S., Sandmann U., Genz A. (2005). Serum concentrations of haloperidol pyridinium metabolites and the relationship with tardive dyskinesia and parkinsonism: A cross-section study in psychiatric patients. Pharmacopsychiatry.

[81] Usuki E., Bloomquist J.R., Freeborn E., Castagnoli K., Van Der Schyf C.J., Castagnoli N. (2002). Metabolic studies on haloperidol and its tetrahydropyridinyl dehydrationproduct (HPTP) in C57BL/6 mouse brain preparations. Neurotox. Res..

[82] Reinke A., Martins M.R., Lima M.S., Moreira J.C., Dal-Pizzol F., Quevedo J. (2004). Haloperidol and clozapine, but not olanzapine, induces oxidative stress in rat brain. Neurosci. Lett..

[83] Ukai W., Ozawa H., Tateno M., Hashimoto E., Saito T. (2004). Neurotoxic potential of haloperidol in comparison with risperidone: Implication of Akt-mediated signal changes by haloperidol. J. Neural Transm..

[84] Saldana M., Bonastre M., Aguilar E., Marin C. (2006). Role of nigral NFkappaB p50 and p65 subunit expression in haloperidol-induced neurotoxicity and stereotyped behavior in rats. Eur. Neuropsychopharmacol..

[85] Mena M.A., de Yébenes J.G. (2006). Drug-induced parkinsonism. Expert Opin. Drug Saf..

[86] Cui X., Pelekanos M., Liu P.-Y., Burne T.H.J., McGrath J.J., Eyles D.W. (2013). The vitamin D receptor in dopamine neurons; its presence in human substantia nigra and its ontogenesis in rat midbrain. Neuroscience.

[87] Liu Y., Li Y.W., Tang Y.L., Liu X., Jiang J.-H., Li Q.-G., Yuan J.-Y. (2013). Vitamin D: Preventive and therapeutic potential in Parkinson’s disease. Curr. Drug Metab..

[88] Eyles D.W., Smith S., Kinobe R., Hewison M., McGrath J.J. (2005). Distribution of the vitamin D receptor and 1 alpha-hydroxylase in human brain. J. Chem. Neuroanat..

[89] Chen T., Yang Y.F., Luo P., Liu W., Dai S.-H., Zheng X.-R., Fei Z., Jiang X.-F. (2013). Homer1 knockdown protects dopamine neurons through regulating calcium homeostasis in an in vitro model of Parkinson’s disease. Cell. Signal..

[90] Ibáñez-Sandoval O., Carrillo-Reid L., Galarraga E., Tapia D., Mendoza E., Gomora J.C., Aceves J., Bargas J. (2007). Bursting in substantia nigra pars reticulata neurons in vitro: Possible relevance for Parkinson disease. J. Neurophysiol..

[91] Dontseva E.A., Trefilova V.V., Popova T.E., Petrova M.M., Al-Zamil M. (2021). Perspectives of personalized approach to prevention and treatment of anticonvulsant-induced osteoporosis via action on vitamin D exchange and VDR expression. Pers. Psychiatry Neurol..

[92] Blandini F., Porter R.H.P., Greenamyre J.T. (1996). Glutamate and Parkinson’s disease. Mol. Neurobiol..

[93] Hurley M.J., Dexter D.T. (2012). Voltage-gated calcium channels and Parkinson’s disease. Pharmacol Ther..

[94] Ogundele O.M., Okunnuga A.A., Fabiyi T.D., Olajide O.J., Akinrinade I.D., Adeniyi P.A., Ojo A.A. (2014). NMDA receptor inhibition and potentiation affects cellular process formation in melanocytes; a model for synaptic denervation in Parkinsonism. Metabol. Brain Dis..

[95] Ogundele O.M., Nanakumo E.T., Ishola A.O., Obende O.M., Enye L.A., Balogun W., Cobham A., Abdulbasit A. (2015). NMDA R/+VDR pharmacological phenotype as a novel therapeutic target in relieving motor-cognitive impairments in Parkinsonism. Drug. Chem. Toxicol..

[96] Cazorla M., de Carvalho F.D., Chohan M.O., Shegda M., Chuhma N., Rayport S., Ahmari S.E., Moore H., Kellendonk C. (2014). Dopamine D2 receptors regulate the anatomical and functional balance of basal ganglia circuitry. Neuron.

[97] Bishnoi M., Chopra K., Kulkarni S.K. (2008). Activation of striatal inflammatory mediators and caspase-3 is central to haloperidolinduced orofacial dyskinesia. Eur. J. Pharmacol..

[98] Voronkov D.N., Khudoerkov R.M., Dovedova E.L. (2013). Changes in neuroglial interactions in the cerebral nigrostriatal structures in a model of dopamine system dysfunction. Zhurnal Nevrol. I Psikhiatrii Im. S.S. Korsakova.

[99] Byron K.Y., Bitanihirwe B.K., Tsung-Ung W.W. (2010). Oxidative Stress in Schizophrenia: An Integrated Approach. Neurosci. Biobehav. Rev..

[100] Drago A., Giegling I., Schäfer M., Hartmann A.M., Friedl M., Konte B., Möller H.-J., De Ronchi D., Stassen H.H., Serretti A. (2013). AKAP13, CACNA1, GRIK4 and GRIA1 genetic variations may be associated with haloperidol efficacy during acute treatment. Eur. Neuropsychopharmacol..

[101] Zhang Y.-M., Wang C.-Y., Zheng F.-C., Gao F.-F., Chen Y.-C., Huang Z.-Q., Xia Z.-Y., Irwin M.G., Li W.-Q., Liu X.-P. (2012). Effects of N-n-butyl haloperidol iodide on the rat myocardial sarcoplasmic re- 635 ticulum Ca(2+)-ATPase during ischemia/reperfusion. Biochem. Biophys. Res. Commun..

[102] Delotterie D., Ruiz G., Brocard J., Schweitzer A., Roucard C., Roche Y., Suaud-Chagny M.-F., Bressand K., Andrieux A. (2010). Chronic administration of atypical antipsychotics improves behavioral and synaptic defects of STOP null mice. Psychopharmacology.

[103] Hasbi A., Fan T., Alijaniaram M., Nguyen T., Perreault M.L., O’Dowd B.F., George S.R. (2009). Calcium signaling cascade links dopamine D1–D2 receptor heteromer to striatal BDNF production and neuronal growth. Proc. Natl. Acad. Sci. USA.

[104] Villalba R.M., Smith Y. (2010). Striatal Spine Plasticity in Parkinson’s Disease. Front. Neuroanat..

[105] Petersen M.S., Bech S., Christiansen D.H., Schmedes A.V., Halling J. (2014). The role of vitamin D levels and vitamin D receptor polymorphism on Parkinson’s disease in the Faroe Islands. Neurosci. Lett..

[106] Peterson A.L., Murchison C., Zabetian C., Leverenz J.B., Watson G.S., Montine T., Carney N., Bowman G.L., Edwards K., Quinn J.F. (2013). Memory, mood, and vitamin d in persons with Parkinson’s disease. J. Parkinsons Dis..

[107] Peterson A.L. (2014). A review of vitamin D and Parkinson’s disease. Maturitas.

[108] Myrianthopoulos N.C., Kurland A.A., Kurland L.T. (1962). Hereditary predisposition in drug-induced parkinsonism. Arch. Neurol..

[109] Richardson M.A., Haugland G., Craig T.J. (1991). Neuroleptic use, parkinsonian symptoms, tardive dyskinesia, and associated factors in child and adolescent psychiatric patients. Am. J. Psychiatry..

[110] Theodoulou G., Milner G., Jumaian A. (2001). Neuroleptics and family history of Parkinson’s diseases: Case report. East. Mediterr. Health J..

[111] Honer W.G., Kopala L.C., Rabinowitz J. (2005). Extrapyramidal symptoms and signs in first-episode, antipsychotic exposed and non-exposed patients with schizophrenia or related psychotic illness. J. Psychopharmacol..

[112] Kalgutkar A.S., Taylor T.J., Venkatakrishnan K., Isin E.M. (2003). Assessment of the contributions of CYP3A4 and CYP3A5 in the metabolism of the antipsychotic agent haloperidol to its potentially neurotoxic pyridinium metabolite and effect of antidepressants on the bioactivation pathway. Drug Metab. Dispos..

[113] Vaiman E.E., Shnayder N.A., Neznanov N.G., Nasyrova R.F. (2021). Candidate genes of the development of antipsychotic-induced parkinsonism in patients with schizophrenia. V.M. Bekhterev Rev. Psychiatry Med. Psychol..

[114] Abdyrakhmanova A.K., Nasyrova R.F. (2022). Pharmacogenetic testing of cytochrome P450 metabolizing enzymes in 28-year-old man with treatment-resistant schizophrenia. Pers. Psychiatry Neurol..

[115] Vaiman E.E., Novitsky M.A., Nasyrova R.F. (2021). Pharmacogenetics of chlorpromazine and its role in the development of antipsychotic-induced parkinsonism. Pers. Psychiatry Neurol..

[116] Vaiman E.E., Shnayder N.A., Novitsky M.A., Dobrodeeva V.S., Goncharova P.S., Bochanova E.N., Sapronova M.R., Popova T.E., Tappakhov A.A., Nasyrova R.F. (2021). Candidate genes encoding dopamine receptors as predictors of the risk of antipsychotic-induced parkinsonism and tardive dyskinesia in schizophrenic patients. Biomedicines.

[117] Vaiman E.E., Shnayder N.A., Neznanov N.G., Nasyrova R.F. (2019). Antipsychotic-induced tardive dyskinesia as a serious adverse effect in the psychopharmacotherapy of schizophrenia. Neurol. Neuropsychiatry Psychosom..

[118] Vaiman E.E., Shnayder N.A., Neznanov N.G., Nasyrova R.F. (2020). Candidate genes involved in the development of antipsychotic-induced tardive dyskinesia in patients with schizophrenia. Neuromuscul. Dis..

[119] Shireen E. (2016). Experimental treatment of antipsychotic-induced movement disorders. J. Exp. Pharmacol..

[120] Ivashchenko D.V., Buromskaya N.I., Shimanov P.V., Deitch D.V., Ryzhykova K.A., Grishina A., Shevchenko Y.S., Sychev D.A. Pharmacogenetics biomarkers of antipsychotics’ safety in adolescents with acute psychotic episode. V.M. Bekhterev Rev. Psychiatry Med. Psychol..

[121] Golimbet V.E., Golov A.K., Kondratyev N.V. (2019). Post-GWAS era in genetics of schizophrenia. V.M. Bekhterev Rev. Psychiatry Med. Psychol..

[122] Abdyrakhmanova A.K., Shnayder N.A., Neznanov N.G., Nasyrova R.F. (2021). Pharmacogenetics of quetiapine. Pers. Psychiatry Neurol..

